# Epigenetic Upregulation of Corticotrophin-Releasing Hormone Mediates Postnatal Maternal Separation-Induced Memory Deficiency

**DOI:** 10.1371/journal.pone.0094394

**Published:** 2014-04-09

**Authors:** Aiyun Wang, Wenying Nie, Haixia Li, Yuhua Hou, Zhen Yu, Qing Fan, Ruopeng Sun

**Affiliations:** Department of Pediatrics, Qilu Hospital, Shandong Univeristy, Jinan, Shandong, P.R. China; Nathan Kline Institute and New York University School of Medicine, United States of America

## Abstract

Accumulating evidences demonstrated that early postnatal maternal separation induced remarkable social and memory defects in the adult rodents. Early-life stress induced long-lasting functional adaptation of neuroendocrine hypothalamic-pituitary-adrenal axis, including neuropeptide corticotrophin-releasing hormone (CRH) in the brain. In the present study, a significantly increased hippocampal CRH was observed in the adult rats with postnatal maternal separation, and blockade of CRHR1 signaling significantly attenuated the hippocampal synaptic dysfunction and memory defects in the modeled rats. Postnatal maternal separation enduringly increased histone H3 acetylation and decreased cytosine methylation in *Crh* promoter region, resulting from the functional adaptation of several transcriptional factors, in the hippocampal CA1 of the modeled rats. Enriched environment reversed the epigenetic upregulation of CRH, and ameliorated the hippocampal synaptic dysfunction and memory defects in the adult rats with postnatal maternal separation. This study provided novel insights into the epigenetic mechanism underlying postnatal maternal separation-induced memory deficiency, and suggested environment enrichment as a potential approach for the treatment of this disorder.

## Introduction

Early postnatal period is extremely critical for the development of normal brain function, and it is vulnerable to the physical injury and adverse early life events. Repeated postnatal maternal separation is one of the most potent stressors to which neonates can be exposed, and may permanently modify neurobiological and behavioral parameters in the adulthood [1]. Early postnatal maternal separation may induce remarkable social and memory defects in the adult rodents. It was recently reported that postnatal maternal separation led to long-lasting hyperactivity of neuroendocrine hypothalamic-pituitary-adrenal (HPA) axis and abnormal passive stress coping and memory, which was associated with the persistent upregulation of the expression of arginine vasopressin due to the sustained DNA hypomethylation of the promoter region of *Avp* in mice [2]. Repeated maternal separation during postnatal days 1–10 significantly lowered the behavioral performance in the contextual fear conditioning test in the rats at postnatal day 60 [3]. Currently the epigenetic mechanism underlying the postnatal maternal separation-induced memory deficiency remains speculative in the rodent model.

Corticotrophin-releasing hormone (CRH), a 41-amino acid neuropeptide derived from a 196-amino acid preprohormone, is expressed and secreted from the central neurons in the paraventricular nucleus and several other brain regions including hippocampus. Hippocampal CRH is generally synthesized and contained within interneurons residing in the pyramidal cell layers of areas CA1 and CA3 [4]. The expression of CRH in several brain regions is appreciably increased in response to the acute or chronic stress [5], [6]. The functional adaptation of stress hormones, including CRH, substantially sculpted the hippocampal glutamatergic strength and synaptic plasticity, and modulated the cognitive function in time- and dose-dependent manner [7]. There exist two subtypes of CRH receptors, CRHR1 and CRHR2, which belong to the G-protein coupled receptor family members [8]. CRHR1 is highly expressed in the hippocampal pyramidal cells [9], and the CRH-CRHR1 signaling is primarily responsible for the stress-impaired hippocampal glutamatergic transmission and memory function [10]. The epigenetic modification of CRH signaling and its involvement in postnatal maternal separation-induced memory deficiency need to be further elucidated.

The effect of enriched environment on brain function was firstly proposed by the observation that the animals with enriched experience in early life had better performance in problem solving at maturity [11], [12]. Extended studies revealed that environmental enrichment significantly improved the capacities of learning and memory, and induced a reversal of learning defects caused by neurological disorders [13], neuronal damage [14], sustained stress [15] or poor maternal parenting [16] in the rodents. Further cellular studies demonstrated that environmental enrichment significantly increased the trafficking of glutamate receptor subunits (NR2A, NR2B, and GluR1) to postsynaptic membrane in the neurons of hippocampus or other brain regions, and facilitated the induction of high-frequency electric stimuli-induced long-term potentiation in the central neurons [17], [18]. Emerging evidences showed that the parental environmental enrichment paradigm remarkably decreased global methylation in the hippocampus of the offspring [19], which was proposed to enhance the brain function. Here the present study investigated the effect of environment enrichment on the memory deficiency induced by postnatal maternal separation and the underlying epigenetic mechanism in the rodent model.

## Materials and Methods

### 1. Animals and Early-life Maternal Separation

Pregnant Sprague-Dawley rats were purchased from the Institutional Center of Experimental Animals. All animal procedures were approved by the Institutional Animal Care and Use Committee of Shandong University (No. 2011-0205), and were carried out in strict accordance with the guidelines of the National Institute of Health.

Postnatal maternal separation was performed as previously reported [2]. Briefly, pups delivered (postnatal day 0 (P0) on day of birth) by timed-pregnant Sprague-Dawley rats were placed, as individual litters, in a clean cage (with heating pad) for 3 h each day on P1–10, having no physical contact with their mothers. Control pups remained undisturbed in the maternal nest throughout. Pups remained with their mothers until weaning at P21.

### 2. Animal Housing (Standard Lab Condition and Enriched Lab Condition)

After weaning at day 21, the rats were housed in either standard lab condition or enriched environment as previously described [17]. The enriched environment was comprised of a spacious cage with various toys, tunnels, platforms, spin wheels. These objects were changed daily to make the environment always novel for rats. During the enrichment period, rats were allowed to explore the enriched environment for 6 hours per day continuously for 8 weeks. Some other rats were group housed under standard lab conditions but were not exposed to the chambers with enriched environment. All experiments were conducted after 8 weeks of enrichment or stand lab condition housing period.

### 3. Morris Water Maze Test

Morris water maze test was performed to assess the spatial learning and memory in the rats of all appropriate groups as previously reported [20]. The animals were 11 weeks old at the time of testing. A circular pool with a diameter of 1.8 meters was filled with opaque water (45 cm deep) at room temperature (24°C). A platform (15 cm in diameter) was submerged 2 cm below the water surface in the center of the target quadrant. Permanent visual cues on the room walls allowed for spatial orientation. A camera mounted above the pool recorded every trial, and animals were tracked using the EthoVision XT software (Noldus Information Technology). Each animal underwent five consecutive training days. Each training session included four trails with different starting position and 15-minute inter-trail intervals. If the animal did not find the platform within 120 seconds it was guided to it. The animals were allowed to sit on the platform for 20 seconds, and then moved back into their home cages. For every trial the time for animal spent to reach the platform was recorded for analysis. On the sixth day, the platform was removed from the tank, and the rats were allowed to swim in the maze for 60 s for a probe trail.

### 4. Novel Object Recognition Test

Novel object recognition test, consisting of three phases of habituation, acquisition trial, and testing trial, was performed in the rats with the previously reported protocol [21]. Rats were habituated in an open-field arena for 20 min on three consecutive days under dim ambient light conditions. The activity of rats was recorded with a video camera. In an acquisition trial, two identical objects were placed in diagonally opposite corners of the chamber 8–9 cm from the walls. A rat was placed at the midpoint between the objects. After allowing 10 min to explore the objects, the rat was returned to the home cage. The testing trail was performed 4 h after the acquisition trail. In the testing trail, one of the objects was replaced with a novel object with the same height and volume but different shape and appearance. For testing, the rat was again placed in the chamber to explore the objects for 3 min. The amount of time spent exploring each object (nose sniffing and head orientation within <1.0 cm) was recorded and evaluated by operators blinded to the treatment. All objects and the test box were cleaned with 70% ethanol between rats to eliminate the odor cues. Novel preference was calculated by the time exploring on the novel object (not presented in the acquisition trial) divided by total exploration time on both novel and familiar objects (presented in the acquisition trial).

### 5. Chromatin Immunoprecipitation (ChIP) and Sequential ChIP Assay

The ChIP assay was performed as previously described with minor modifications [22]. The hippocampal CA1 tissues were collected and fixed in 1% formaldehyde for 15 min at room temperature. Chromatin was solubilized and sonicated on ice 8×10 s, followed by 1 min cooling on ice to produce fragments of approximately 300–500 bp with a BioRuptor (Diagenode). Sonicated samples were centrifuged at 14,000 g for 10 min, and the supernatants were collected. A tenth of the lysates was saved as input DNA. The samples were precleared with salmon sperm DNA/protein A agarose beads. The polyclonal antibody against acetylated histone H3, HDAC2 (1∶100, Millipore), MeCP2 or DNMT1 antibody (1∶100, Cell signaling) was added to each sample and incubated overnight at 4°C with gentle mixing. Rabbit IgG was used as the negative control, and monoclonal anti-RNA polymerase II antibody (1∶100, Millipore) was used as the positive control. Immunocomplexes were enriched by salmon sperm DNA/protein A agarose beads. After extensive wash with low salt, high salt, LiCl and TE buffers (Millipore), the DNA/protein complexes was eluted by adding 200 μl freshly made elution buffer (0.1 M NaHCO_3_/1% SDS), vortexing, and 10 min rotation at RT. The crosslinks were reversed by adding 8 μl of 5 M NaCl incubated at 65°C overnight. The DNA fragments were purified with phenol-chloroform extraction followed by acid ethanol precipitation.

Sequential ChIP assay was performed as described previously [23], [24]. The hippocampal CA1 tissues were cross-linked with 1.0% formaldehyde and subsequently processed as described above. Polyclonal antibody against MeCP2 was used in the first round of ChIP. The salmon sperm DNA/protein A agarose beads with immunocomplexes were incubated with equal volume of 10 mM DTT for 30 minutes at 37°C, centrifuged and supernatants containing precipitated chromatin fragments were collected into new tubes. The eluted samples were diluted 10 times with IP buffer and 20% of the sample was saved as input control. Second round of ChIP was then performed with antibody against HDAC2 or DNMT1 according to the protocol as described above. Rabbit IgG was used as negative control.

Real-time PCR was performed to amplify approximately 200 bp fragments (−247 bp to −35 bP) within the *Crh* transcriptional control region. Primer sets were used as following: *Crh* (5′- TTCCATTTTAGGGCTCGTTG-3′, 5′- CGACCCTCTTCAGAAAGCAC-3′), *Gapdh* (5′-AGACAGCCGCATCTTCTTGT-3′, 5′-CTGCGGGAGAAGAAAGTCAG-3′). The ChIP signal was analyzed as the following method in the handbook provided by the manufacturer: ΔCt_[normalized ChIP_ = (Ct_[ChIP]_ − (Ct_[Input]_ − Log_2_(input dilution factor))); and ChIP/Input ratio was calculated as 2 ^(−ΔCt [normalized ChIP])^.

### 6. Methylated DNA Immunoprecipitation (MeDIP) Assay

The MeDIP assay was performed as previously described with minor modification [25]. Briefly, hippocampal CA1 tissue was homogenized in the lysis buffer and genomic DNA was sonicated on ice 8×10 s. Sonicated samples were centrifuged at 14,000 g for 10 min, and the supernatants were collected. The polyclonal antibody against 5-methylcytosine (1∶100, Millipore) was added to each sample and incubated overnight at 4°C with gentle mixing. The DNA-antibody complex was enriched with protein A agarose beads. DNA fragments in the input and pulled-down fractions were then purified with phenol-chloroform extraction followed by acid ethanol precipitation. Real-time PCR was performed to amplify about 200-bp segments corresponding to CpG sites within *Crh* promoter region. Primer sets were used as following: *Crh* (5′- TTCCATTTTAGGGCTCGTTG-3′, 5′- CGACCCTCTTCAGAAAGCAC-3′), *Gapdh* (5′-AGACAGCCGCATCTTCTTGT-3′, 5′-CTGCGGGAGAAGAAAGTCAG-3′). Amplifications were run in triplicate, and the PCR data were analyzed as above.

### 7. Quantitative Real-Time (RT) PCR

Hippocampal CA1 tissue was sampled, and the total RNA was extracted with TRIzol reagent (Invitrogen). Total cDNA was synthesized using SuperScript III Reverse Transcriptase (Invitrogen), and real-time PCR was performed in triplicate. The sequences of the primers to measure CRH mRNA were: 5′- CTCTCTGGATCTCACCTTCCAC-3′ and 5′- CTAAATGCAGAATCGTTTTGGC-3′. The primers for GAPDH mRNA were: 5′- AGACAGCCGCATCTTCTTGT-3′ and 5′- CTTGCCGTGGGTAGAGTCAT-3′. Fold change was calculated using the ΔΔCT method, and compared between the appropriate groups.

### 8. Protein Extraction and Immunoblotting

The protein extraction and immunoblotting was performed generally following the previous reports [26]. The hippocampus CA1 tissues were collected and lysed in ice-cold lysis buffer containing 55 mM Tris–Cl, 145 mM NaCl, 0.01 mM NaN_3_, 100 μg/ml phenylmethyl sulfonyl fluoride, 1 μg/ml aprotinin, 1% Triton X-100 and proteinase inhibitor cocktail. Cytoplasmic and nuclear protein were extracted and separated with SDS - polyacrylamide gel (7.5%) electrophoresis followed by blotting to a nitrocellulose membrane. The blots were incubated overnight at 4°C with the primary antibodies as following: polyclonal anti-MeCP2 primary antibody (1∶1000; Cell signaling), polyclonal anti-phosphorylated MeCP2 (serine 421) antibody (1∶1000; ECM Biosciences) and monoclonal anti-β-actin antibody (1∶2000; Santa Cruz Biotechnology). After extensive wash, the membranes were incubated with horseradish peroxidase-conjugated anti-mouse and anti-rabbit IgG antibody (1∶10,000; Jackson ImmunoResearch Laboratories Inc.). The immunoreactivity was detected using enhanced chemiluminescence (ECL Advance Kit; Amersham Biosciences). The intensity of the bands was captured digitally and analyzed quantitatively with ImageJ software. The immunoreactivity of all proteins was normalized to that of β-actin.

### 9. Enzyme Linked Immunosorbent Assay (ELISA)

Enzyme linked immunosorbent assay was performed to measure the CRH level in the hippocampal CA1 tissues of the rats in appropriate groups. Commercial ELISA kits (Kamiya Biomedical Company, Seattle, USA) were applied following the instructions provided by the manufacturer. The detect range was 1–1000 pg/ml. The absorbance was measured on a microplate reader at 450 nm, and the concentrations of CRH were calculated from a standard curve for each sample.

### 10. Hippocampal Slice Preparation and Whole-cell Recordings

Hippocampal slices were prepared and the whole-cell recording was performed on the CA1 neurons as previously described [27]. The rats were deeply anesthetized with inhalation of halothane, and the brain was removed quickly. The coronal brain slices containing hippocampal CA1 were cut with a vibratome (Technical Products International, St. Louis, MO), and incubated in Krebs solution (containing: 117 mM NaCl, 3.6 mM KCl, 1.2 mM MgCl_2_, 2.5 mM CaCl_2_, 1.2 mM NaH_2_PO_4_, 11 mM glucose, and 25 mM NaHCO_3_, bubbled with 95% O_2_ and 5% CO_2_) at 34°C for at least 1 h before the recording was performed.

Hippocampal CA1 neurons were visualized using an upright microscope with infrared illumination (BX50WI; Olympus, Tokyo). Whole-cell voltage-clamp recordings were performed using an Axopatch 700B amplifier (Molecular Devices) with 2–4 MΩ glass electrodes containing the following internal solution (in mM): cesium gluconate, 125; NaCl, 5; MgCl_2_ 1.0; EGTA, 0.5; Mg-ATP, 2; Na_3_GTP, 0.1; HEPES, 10; guanosine 5-*O*-(2-thiodiphosphate) 1; lidocaine *N*-ethyl bromide (QX314), 10; pH 7.3; 290–300 mOsmol. A seal resistance of ≥2 GΩ and an access resistance of 15–20 MΩ were considered acceptable. The series resistance was optimally compensated by ≥70% and constantly monitored throughout the experiments. The membrane potential was held at −60 mV throughout the experiment. Schaffer collateral–commissural fibers were stimulated by concentric bipolar electrode, and the excitatory postsynaptic currents (EPSCs) were recorded in the CA1 area in the presence of bicuculline (30 μM). The evoked EPSCs were filtered at 2 kHz, digitized at 10 kHz, and acquired and analyzed using pCLAMP 9.2 software (Molecular Devices). The amplitude of the EPSCs was monitored for a baseline period of at least 15 min. If synaptic transmission was stable (<15% change in EPSC amplitude over 15 min), long-term potentiation (LTP) was induced by a single high-frequency electric stimuli train (HFS, 100 Hz for 1 s) [28]. All electrophysiological experiments were performed at room temperature.

### 11. Compounds and Data Analysis

All chemicals and reagents were purchased from Sigma-Aldrich (St. Louis, MO) or Tocris Bioscience (Ellisville, MO), unless specified otherwise. All data were presented as means ± SEM. For analysis of immunoblotting data, differences between groups were compared by Student’s t-test or ANOVA followed by Fisher’s PLSD post hoc analysis. The electrophysiological and behavioral data were compared with two-way ANOVA with repeated measurements. The criterion for statistical significance was *P*<0.05. Statistical tests were performed with SPSS 13.0 (SPSS, USA).

## Results

### 1. Maternal Separation Impaired Hippocampal Synaptic Plasticity and Memory in the Adult Rats

**Figure 1:**
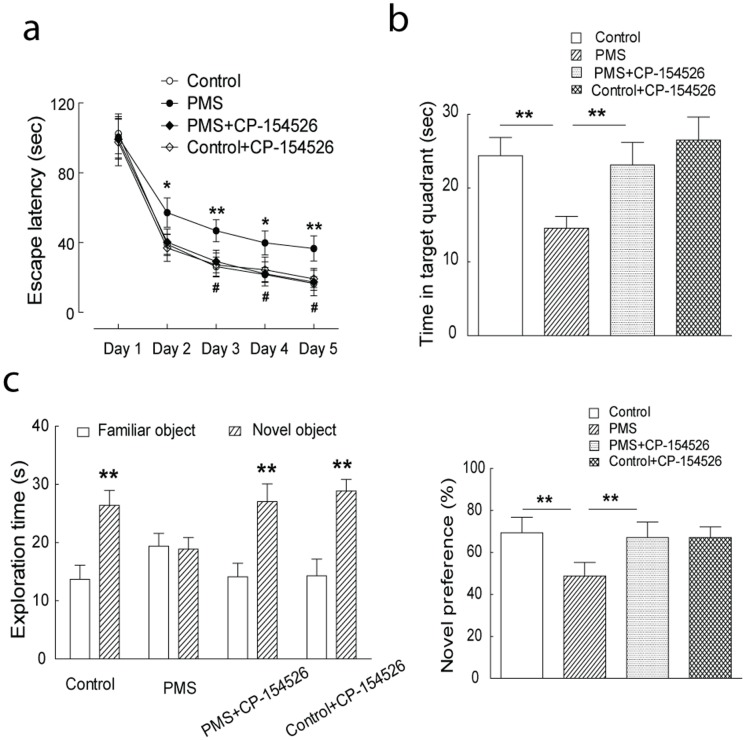
Blockade of CRHR1 signaling by CP-154,526 (10 mg/kg, i.p. for 6 days) significantly improved the behavioral performance in the rats with postnatal maternal separation (PMS). (A and B): The rats with postnatal maternal separation had extended escape latencies in Morris water maze test (F_(1,18)_ = 10.37, n = 9 and 11 rats in each group, P<0.01), which was mitigated by injection of CP-154,526 (F_(1,18)_ = 9.29, n = 11 and 9 rats in each group, P<0.01). These modeled rats also spent less time in the target quadrant in the probe trail, which was mitigated by injection of CP-154,526. (C): The significantly decreased preference to the novel object was observed in the rats with postnatal maternal separation, which was ameliorated by injection of CP-154,526. N  = 9–11 rats per group, *, P<0.05; **, P<0.01; #, P<0.05; ##, P<0.01.

Upon postnatal 11 weeks, no appreciable physical change, including body weight and general physical activities, was observed in the rats with postnatal maternal separation, compared to those in the control group. Morris water maze test and novel object recognition test were performed to evaluate the memory function in the rats with postnatal maternal separation. As shown in Fig. 1A, significantly extended escape latencies in the water maze test were observed in these rats with postnatal maternal separation. Significantly decreased time spent in the target quadrant was also observed in the modeled rats (Fig. 1B). Consistently, the rats with postnatal maternal separation spent less time exploring the novel object, when compared to the control rats (Fig. 1C). These results suggested substantial defects in the learning and memory in the adult rats with postnatal maternal separation.

**Figure 2:**
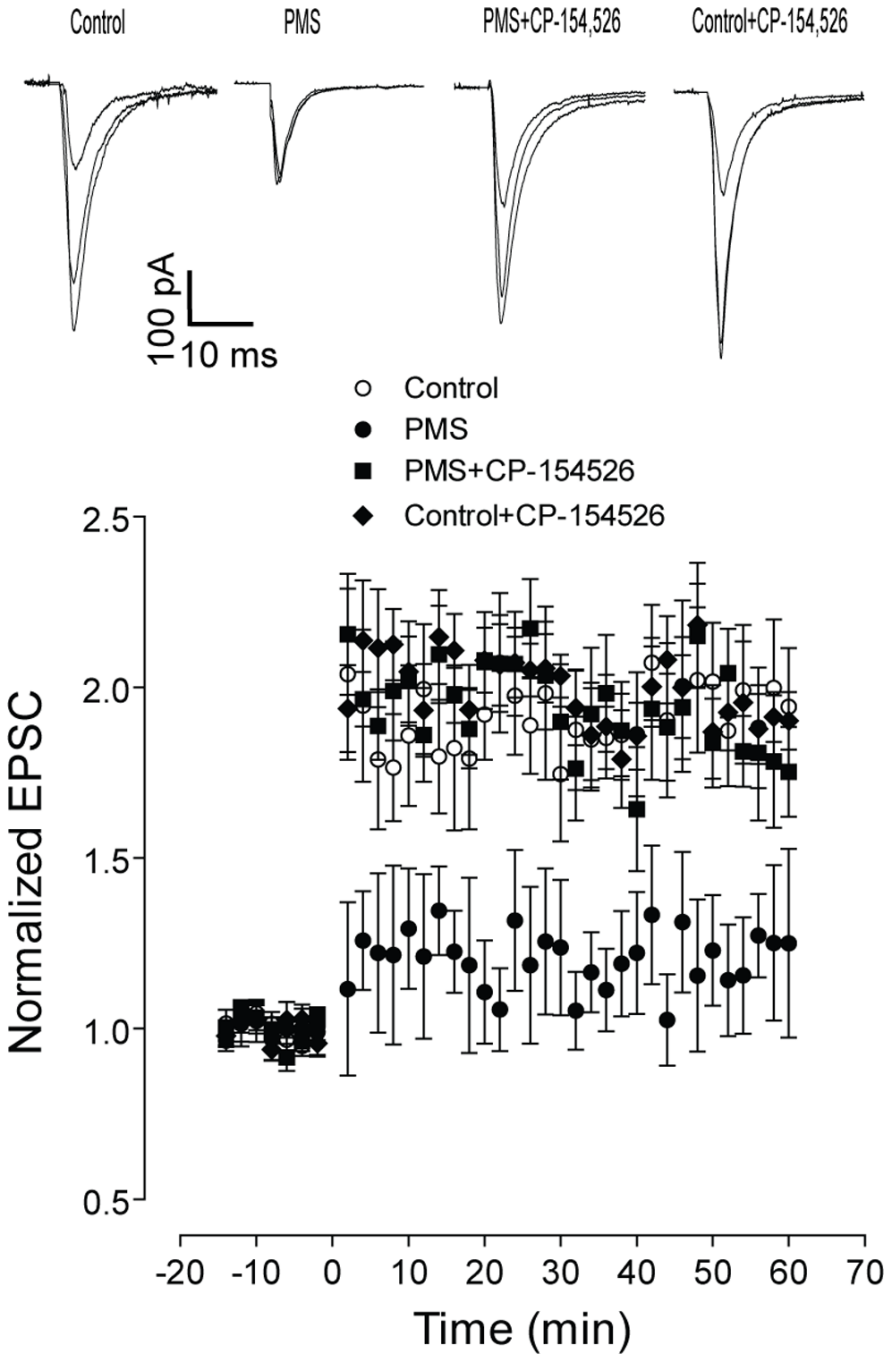
Blockade of CRHR1 signaling by CP-154,526 (10 mg/kg, i.p. for 6 days) significantly improved the high-frequency electric stimuli-induced LTP in hippocampal CA1 of the rats with postnatal maternal separation. Significantly impaired hippocampal LTP was observed in the rats with postnatal maternal separation (F_(1,19)_ = 15.22, n = 9 and 12 neurons in each group, P<0.01), which was mitigated by injection of CP-154,526 (F_(1,20)_ = 11.24, n = 10 and 12 neurons in each group, P<0.01). Representative traces of evoked EPSCs in hippocampal CA1 neurons were presented. N = 9–12 neurons in each group.

Next, the adaptation of glutamatergic synaptic plasticity was investigated in the hippocampal CA1 of the rats with postnatal maternal separation. The evoked EPSCs in hippocampal CA1 neurons were recorded in the presence of bicuculline (30 μM). The evoked EPSCs were completely abolished by the perfusion of APV (30 μM) and CNQX (30 μM), confirming its glutamatergic component. As shown in Fig. 2, HFS - induced LTP was significantly impaired in the rats with postnatal maternal separation, compared to that in the control group. This indicated an impaired hippocampal glutamatergic synaptic plasticity in the rats with postnatal maternal separation.

### 2. Altered Expression of CRH Underlay Maternal Separation-induced Abnormalities in the Adult Rats

**Figure 3:**
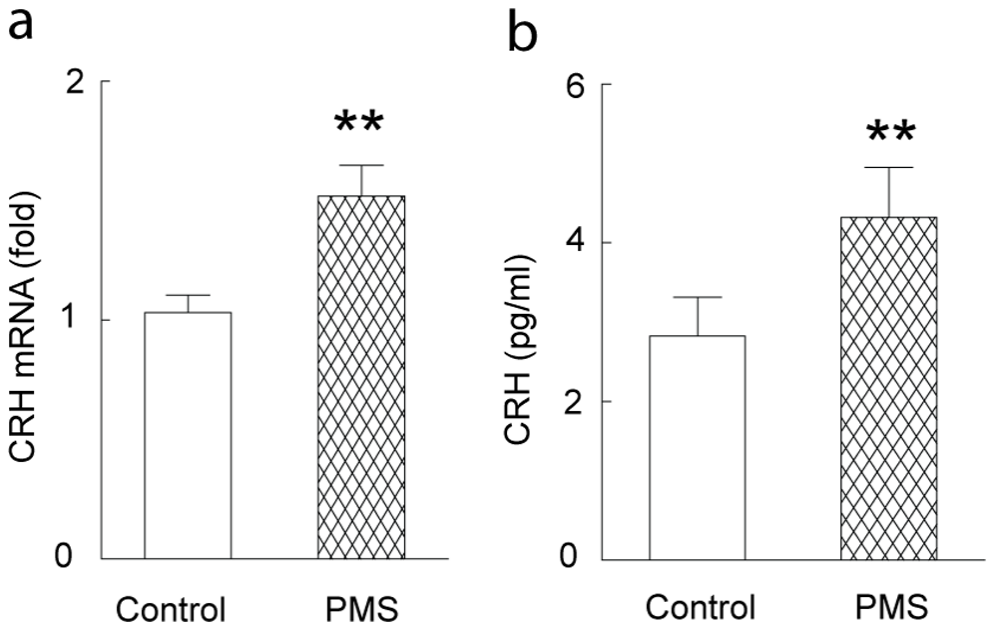
Significantly increased mRNA (A) and protein expression (B) of the CRH was observed in the hippocampal CA1 of the adult rats with postnatal maternal separation. N  = 7–8 rats in each group, **, P<0.01.

Previous studies demonstrated significant alteration of the activity of HPA axis including CRH in the rodents with early life stress [2]. Altered activity of HPA axis, including CRH, substantially contributed to the impaired synaptic plasticity and memory function in the rodents with sustained stress. Here, we examined the change of the CRH in the hippocampal CA1 area in the rats with postnatal maternal separation. As shown in Fig. 3, significantly increased expression, along with the upregulated mRNA level, of the CRH was observed in the hippocampal CA1 of the modeled rats, compared with those in the control rats. These suggested a potential involvement of hippocampal CRH in the glutamatergic synaptic dysfunction and memory deficiency in the rats with postnatal maternal separation.

Next, the CRHR1-mediated signaling was inhibited by the systemic administration of CRHR1 antagonist, CP-154,526 (10****mg/kg, i.p. for 6 days prior to the test) [29], and its effects on hippocampal synaptic plasticity and memory function were investigated in the rats with postnatal maternal separation. As shown in Fig. 2, inhibition of CRHR1 signaling by CP-154,526 significantly recovered the HFS-induced LTP in hippocampal CA1 neurons of the rats with postnatal maternal separation, while it did not have obvious effect in the control rats. In the Morris water maze test, blockade of the CRHR1 signaling significantly shortened the escape latencies and increased the time spent in the target quadrant in the rats with postnatal maternal separation (Fig. 1A and 1B). Consistently, inhibition of CRHR1 signaling significantly increased the time for the rats spent exploring the novel object in the modeled rats (Fig. 1C). Note that inhibition of CRHR1 signaling did not remarkably alter the behavioral performance in the Morris water maze test and novel object recognition test in the control rats (Fig. 1). These results suggested that upregulation of CRH signaling, *via* the receptor CRHR1, mediated the maternal separation-induced hippocampal glutamatergic synaptic dysfunction and memory deficiency in the rats.

### 3. Epigenetic Mechanism Underlying the Upregulation of CRH Induced by Maternal Separation

**Figure 4:**
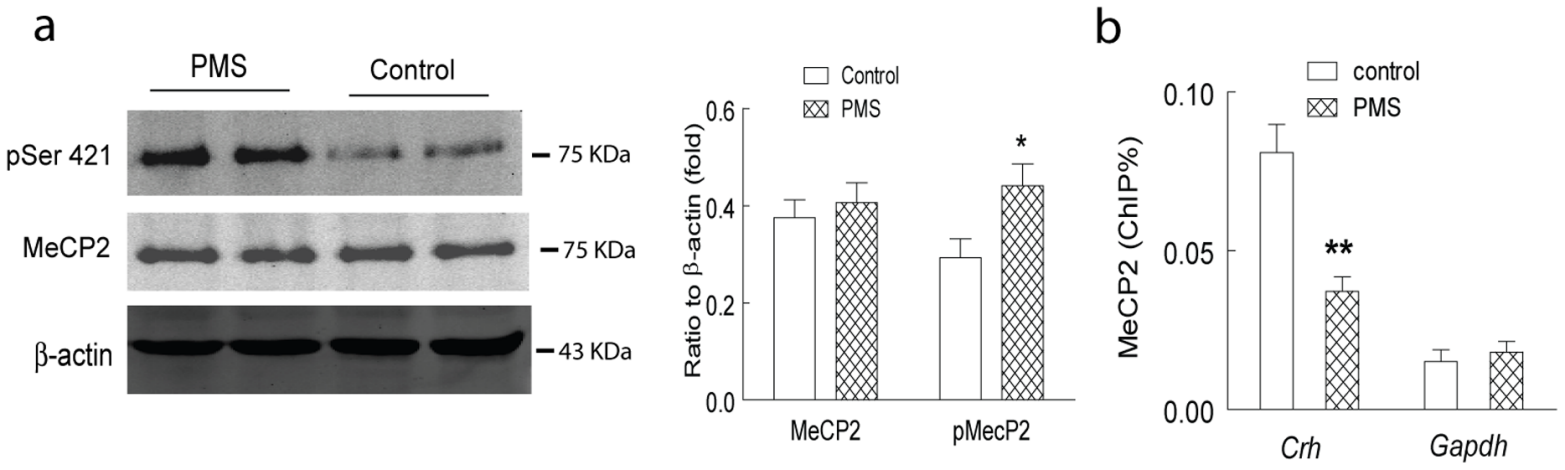
Significantly decreased occupancy of the MeCP2 in the promoter region of *Crh* was observed in the hippocampal CA1 of the rats with postnatal maternal separation (PMS). A: significantly increased phosphorylation (serine 421) of MeCP2, but not the total MeCP2, was observed in the hippocampal CA1 of the rats with postnatal maternal separation (N = 7–8 per group). B: Significantly decreased occupancy of the MeCP2 in the promoter region of *Crh,* but not *Gapdh* in the hippocampal CA1 of the rats with postnatal maternal separation (N = 7–8 per group). *, P<0.05; **, P<0.01.

Methyl CpG binding protein 2 (MeCP2), a transcriptional factor suppressing the expression of target genes, generally binds to the methylated CpG sites in the promoter region, and recruits several other transcriptional factors to modify the histone acetylation and the maintenance of DNA methylation in the promoter region of target genes. Phosphorylation of different serine residues may modify the association of the MeCP2 with the target gene, and modulate the glutamatergic synaptic plasticity and memory. Here, we firstly explored the expression and phosphorylation (serine residue 421) of MeCP2 in the hippocampal CA1 of the rats with postnatal maternal separation. As shown in Fig. 4A, significantly increased phosphorylation at serine 421 of MeCP2 was observed in the hippocampal CA1 of the rats with postnatal maternal separation, although the expression of total MeCP2 was not altered. Further ChIP results also revealed a decreased occupancy of the MeCP2 in the promoter region of *Crh,* but not *Gapdh* (Fig. 4B). These results implied the involvement of MeCP2 in the upregulation of hippocampal *Crh* expression in the rats with postnatal maternal separation.

**Figure 5:**
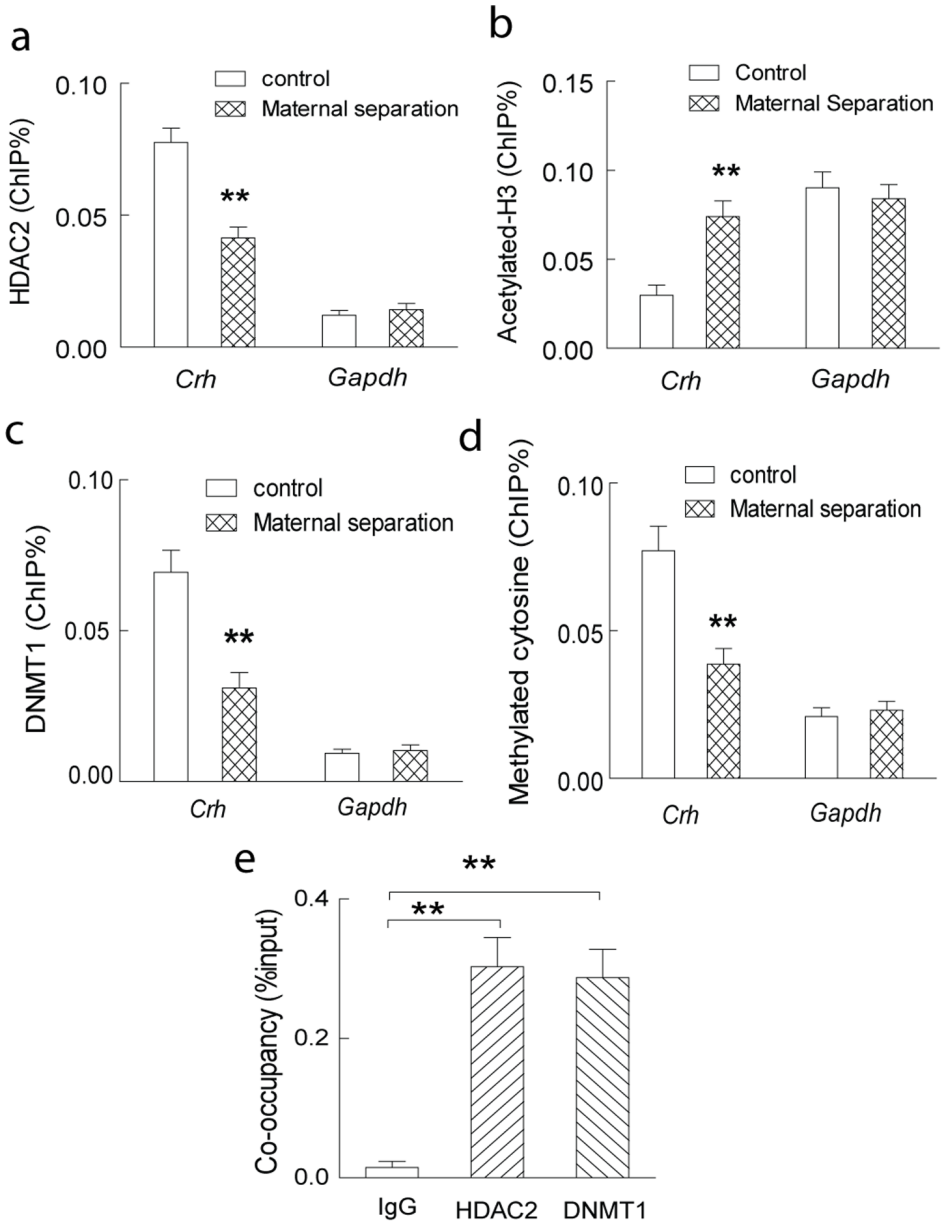
Increased histone H3 acetylation and decreased cytosine methylation in *Crh* promoter region in hippocampal CA1 of the rats with postnatal maternal separation (PMS). The decreased occupancy of HDAC2 (A) and increased histone H3 acetylation (B) were observed in the *Crh*, but not *Gapdh*, promoter region in the hippocampal CA1 of the rats with maternal separation. The decreased occupancy of DNMT1 (C) and decreased methylation of 5′-cytosine (D) were also observed in the *Crh*, but not *Gapdh*, promoter region in the hippocampal CA1 of these modeled rats. (E) MeCP2 was co-localized with HDAC2 and DNMT1 in *Crh* promoter in hippocampal CA1. Sequential ChIP with antibodies against HDAC2 or DNMT1 was performed on chromatin fragments precipitated by using the anti-MeCP2 antibody in the hippocampal CA1 lysates in the control rats. N  = 7–8 per group; **, P<0.01.

Transcriptional repressor HDAC2, recruited to the promoter region by MeCP2, may decrease the histone acetylation in the promoter region, and suppress the expression of target gene. Here, the ChIP study with HDAC2 antibody revealed that the occupancy of HDAC2 in the *Crh*, but not *Gapdh*, promoter region was significantly decreased in the hippocampal CA1 of the rats with maternal separation (Fig. 5A). Consequently, significantly increased histone H3 acetylation was also observed in the *Crh*, but not *Gapdh*, promoter region in the hippocampal CA1 of these modeled rats (Fig. 5B).

Meanwhile, MeCP2 may also partner with the DNA methyltransferase 1 (DNMT1) to form a transcriptional repressor complex, which maintaining the 5′-cytosine methylation in the promoter region of target genes [30], [31]. Here, the present study revealed that the occupancy of DNMT1 in the *Crh*, but not *Gapdh*, promoter region was significantly decreased in the hippocampal CA1 of the rats with postnatal maternal separation (Fig. 5C). Thereafter, significantly decreased methylation of 5′-cytosine was also observed in the *Crh*, but not *Gapdh*, promoter region in the hippocampal CA1 of these modeled rats (Fig. 5D).

Next sequential ChIP assay was used to test whether there was any co-occupancy between MeCP2 and HDAC2 or DNMT1 complex on *Crh* promoter in hippocampal CA1 tissue. Chromatin fragments precipitated by anti-MeCP2 antibody were subjected to second round ChIP with antibodies against HDAC2 or DNMT1, respectively. Significant amount of chromatin containing *Crh* promoter region was enriched by the HDAC2 or DNMT1 antibody in the sequential ChIP study (Fig. 5E). It was notable that the amount of chromatin containing *Crh* promoter precipitated by HDAC2 or DNMT1 antibody in the sequential ChIP studies (Fig. 5E) was significantly higher than that enriched by HDAC2 or DNMT1 antibody alone in the single-round ChIP studies (Fig. 5A and 5C), which indicating the co-localization of MeCP2 with transcriptional factors HDAC2 and DNMT1 in the promoter region of *Crh* in the hippocampal CA1 in control rats. Considering the function of HDAC2 and DNMT1 as transcriptional repressor, these results implied that the histone hyperacetylation and DNA hypomethylation mediated by MeCP2 and associated transcriptional factors might underlie the upregulation of hippocampal *Crh* expression in the rats with postnatal maternal separation.

### 4. Enriched Environment Suppressed CRH Expression, and Ameliorated the Abnormalities Induced by Maternal Separation

**Figure 6:**
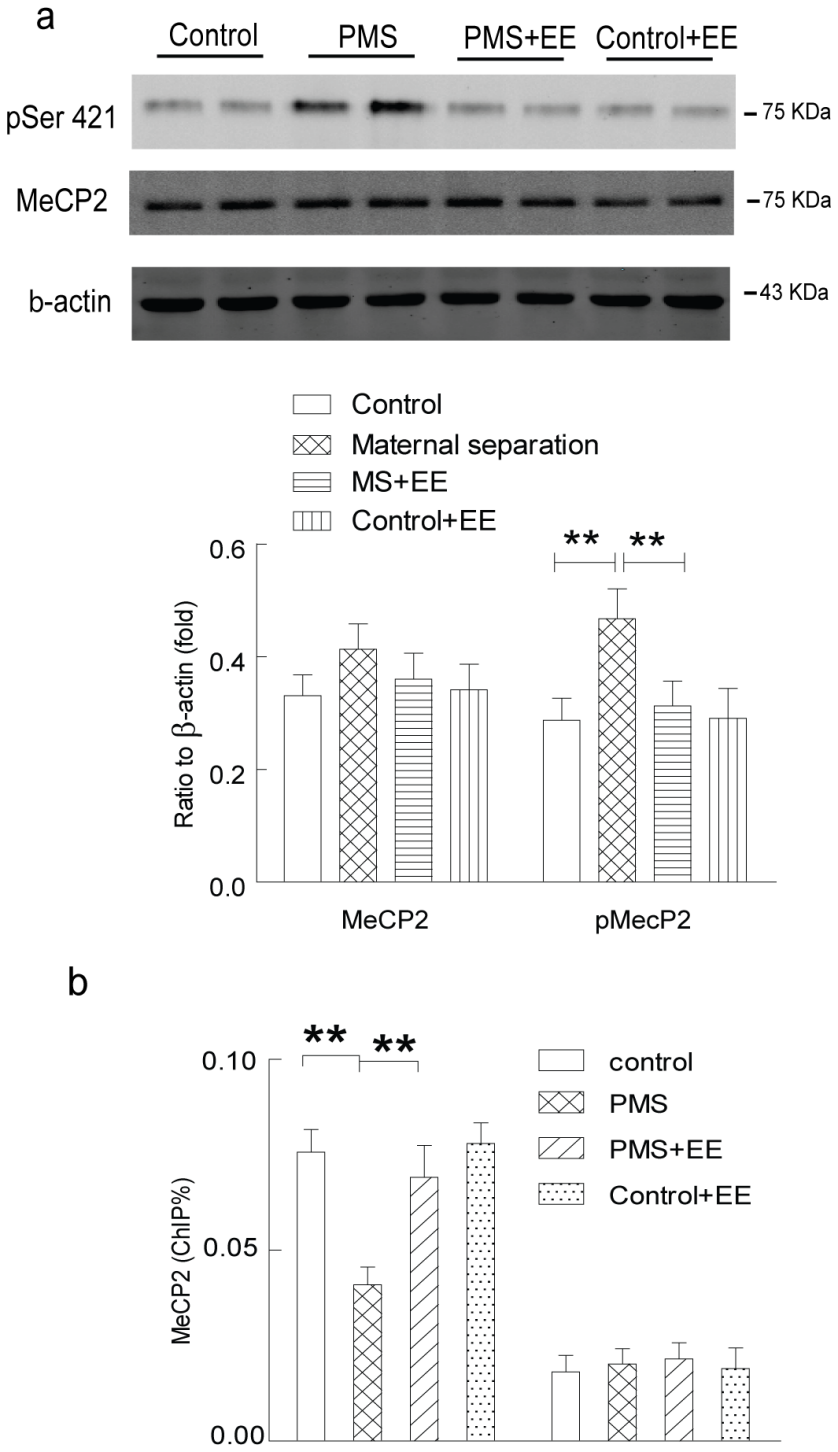
Enriched environment significantly decreased the phosphorylation (serine 421) of MeCP2 (A), and increased the occupancy of MeCP2 in the *Crh*, but not *Gapdh*, promoter region (B) in the hippocampal CA1 in the rats with postnatal maternal separation. N  = 7–8 per group; **, P<0.01.

**Figure 7:**
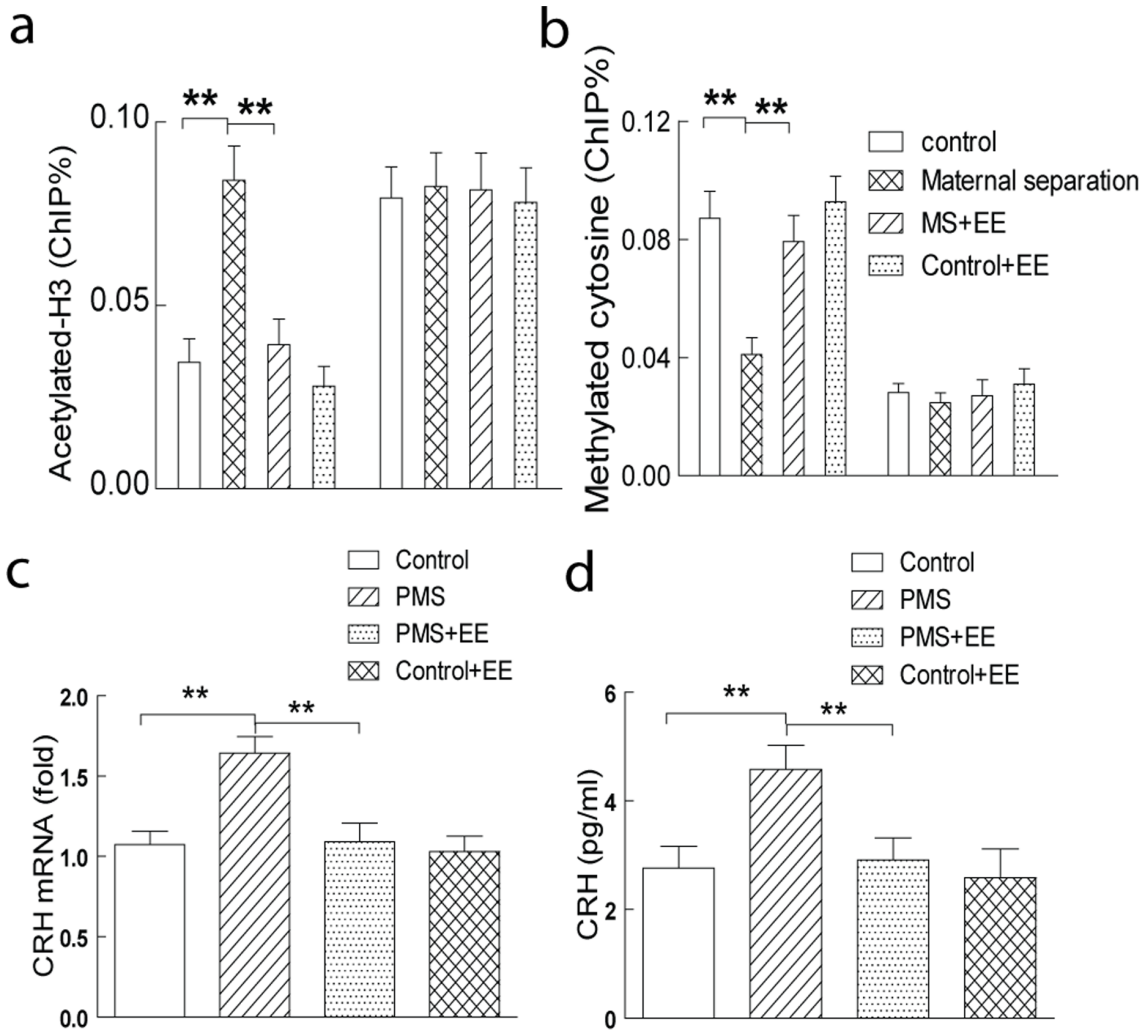
Enriched environment suppressed the upregulation of hippocampal CRH in the rats with postnatal maternal separation. Enriched environment decreased histone H3 acetylation (A), and increased 5′-cytosine methylation (B) in the *Crh*, but not *Gapdh*, promoter region in the hippocampal CA1 in the modeled rats. Enriched environment also significantly suppressed the upregulation of the mRNA (C) and protein expression (D) of CRH in the rats with postnatal maternal separation. N  = 7–8 per group; **, P<0.01.

Previous studies reported that enriched environment remarkably increased the hippocampal synaptic plasticity, and enhanced the memory in the rodents. Here, we investigated whether environmental enrichment recovered the epigenetic upregulation of CRH expression, glutamatergic dysfunction and memory deficiency induced by postnatal maternal separation. Firstly, enriched environment significantly reduced the phosphorylation (serine 421) of MeCP2 (Fig. 6A), and increased the occupancy of MeCP2 in the *Crh*, but not *Gapdh*, promoter region (Fig. 6B) in the hippocampal CA1 in the rats with postnatal maternal separation. We further found that environmental enrichment also decreased hippocampal histone H3 acetylation (Fig. 7A) and increased 5′-cytosine methylation (Fig. 7B) in the *Crh*, but not *Gapdh*, promoter region in the modeled rats. Consistently, enriched environment significantly suppressed the upregulation of the mRNA and protein expression of CRH in the rats with postnatal maternal separation (Fig. 7C and D). These results indicated that enriched environment reversed the epigenetic upregulation of hippocampal CRH induced by the postnatal maternal separation.

**Figure 8:**
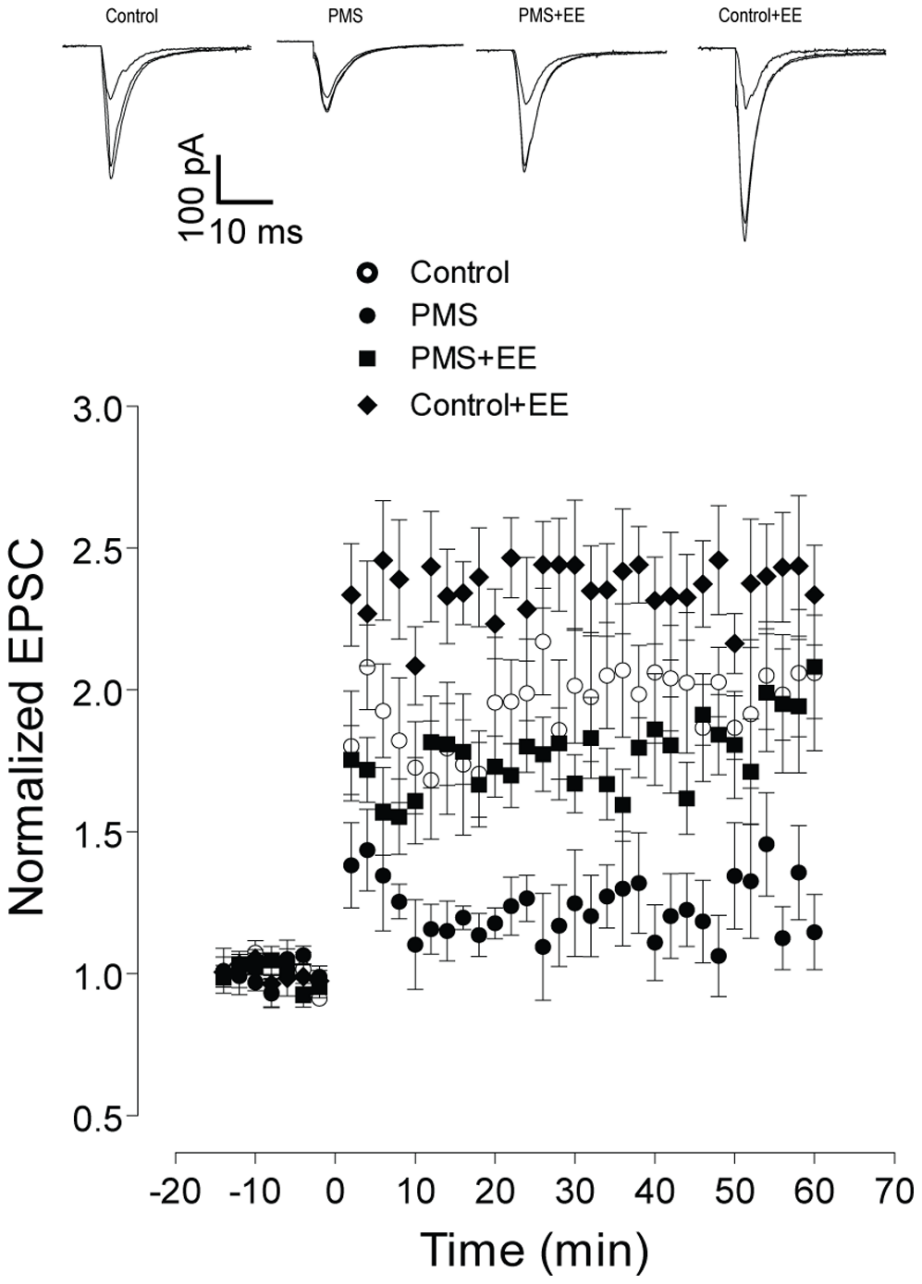
Enriched environment significantly ameliorated the hippocampal HFS-induced LTP impaired by the postnatal maternal separation (F_(1,20)_ = 10.62, n = 10 and 12 neurons in each group, P<0.01), while it also induced marginal increase of hippocampal LTP in the control rats (F_(1,18)_ = 7.46, n = 9 and 11 neurons in each group, P<0.05). Representative traces of evoked EPSCs in hippocampal CA1 neurons were presented. N = 9–12 neurons in each group.

**Figure 9:**
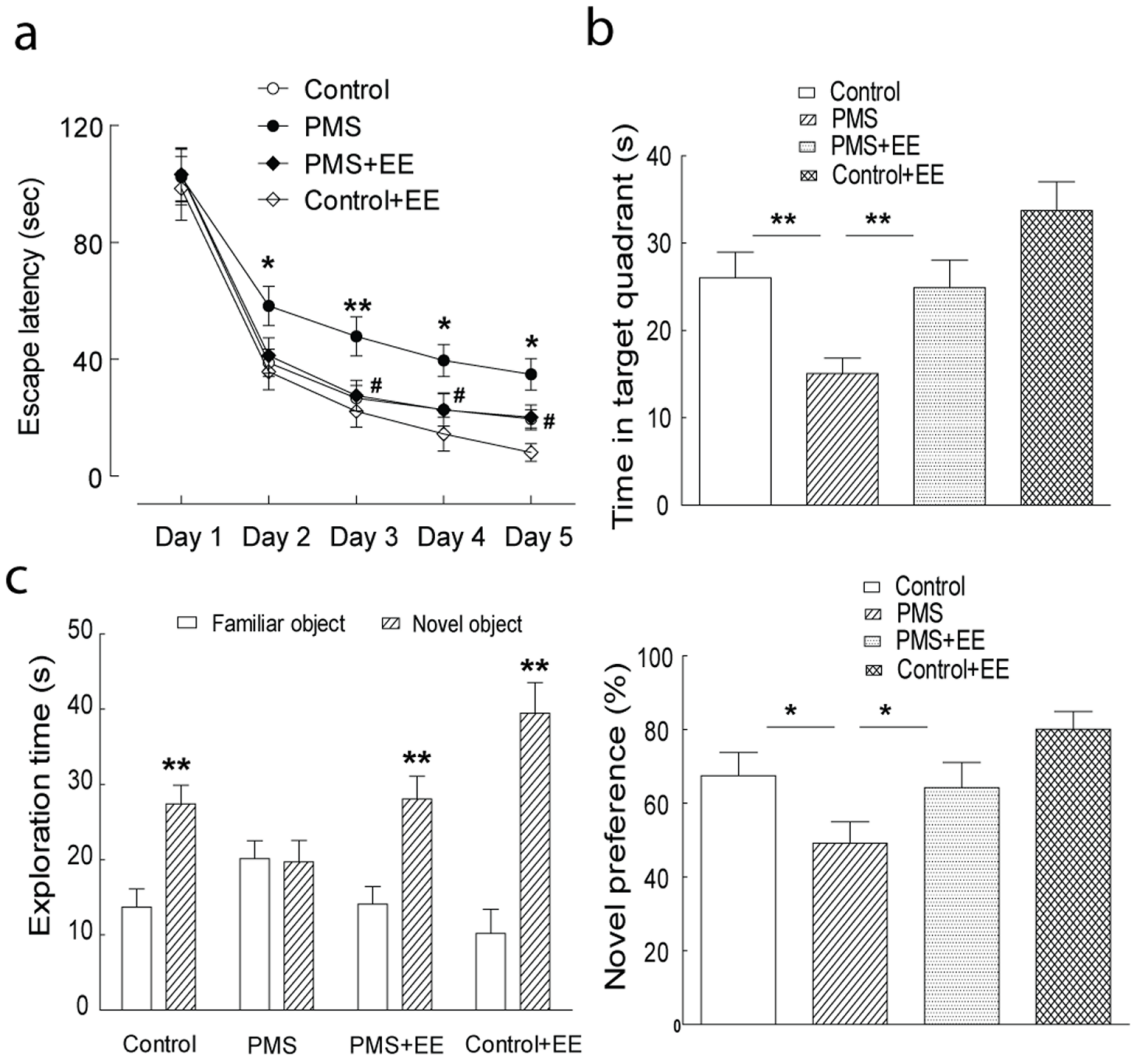
Enriched environment improved the behavioral performance in Morris water maze and novel object recognition tests. (A and B): Enriched environment significantly shortened the escape latencies (F_(1,20)_ = 10.27, n = 10 and 12 rats per group, P<0.01). and increased the time spent in the target quadrant in the rats with postnatal maternal separation (N = 9–12 per group). (C) Enriched environment significantly increased the time for the rats spent exploring the novel object in the modeled rats (N = 9–11 per group). Note that enriched environment induced increase of the behavioral performance in the Morris water maze test (F_(1,19)_ = 4.61, n = 9 and 12 rats per group, P<0.05)and novel object recognition test in the control rats. *, P<0.05; **, P<0.01; #, P<0.05; ##, P<0.01.

Further electrophysiological studies found that enriched environment significantly improved the hippocampal HFS-induced LTP in the rats with postnatal maternal separation, while it also enhanced hippocampal LTP in the control rats (Fig. 8). In the Morris water maze test, enriched environment significantly shortened the escape latencies and increased the time spent in the target quadrant in the rats with postnatal maternal separation (Fig. 9A and 9B). Consistently, in these modeled rats enriched environment significantly increased the time for the rats spent exploring the novel object (Fig. 9C). Note that enriched environment induced marginal increase of the behavioral performance in the Morris water maze test and novel object recognition test in the control rats (Fig. 9). These results raised the possibility that enriched environment might, *via* epigenetic suppression of CRH, mitigate the postnatal maternal separation-induced hippocampal glutamatergic synaptic dysfunction and memory deficiency in the rats.

## Discussion

Increasing evidences demonstrated that postnatal maternal separation substantially induced the functional adaptation of transcriptional factors, and modified the expression of specific proteins to mediate the enduring alteration of social and memory behaviors in the rodents [1], [32]. In Balb/c mice, infant maternal separation decreased the expression of HDACs 1, 3, 7, 8, and 10, and increased acetylation of histone H4 in the forebrain neocortex in adulthood [32]. It was also reported that postnatal maternal separation induced significant hypomethylation of DNA and dissociation of phosphorylated MeCP2 in the promoter region of *Avp*, thus contributing to the sustained hypersecretion of cortisone in the brain [2]. Significantly increased histone acetylation in the promoter regions of *Arc* and *Egr1*, two immediate early genes underlying experience-induced synaptic plasticity, was also recently observed in the hippocampus of mice with maternal separation [33]. Dynamic alteration of hippocampal histone methylation at the *Bdnf* IV promoter and BDNF expression, concomitant with impairments on hippocampal-dependent cognitive tasks, were also observed in the rats with postnatal maternal separation [34]. Consistent evidences also demonstrated that other early life stress from maltreatment or abuse induced substantial modification of methylation patterns at specific loci of the genomic DNA, which in turn permanently altered gene expression in the brain and induced alteration of social behavior in the adult rodents and humans [35]–[38]. In the present study, postnatal maternal separation decreased the occupancy of transcriptional factors (MeCP2, HDAC2 and DNMT1), which leading to the increased histone H3 acetylation and decreased cytosine methylation, in the promoter region of *Crh*, and consequently impaired hippocampal synaptic plasticity and behavioral performance in the cognitive tasks. These results provided further evidence for the postnatal maternal separation – induced lasting epigenetic modification at specific loci of the genomic DNA in the hippocampus.

Epigenetic mechanism was critically involved in the modulation of hippocampal synaptic plasticity and memory function in the rodents [39], [40]. Functional adaptation of MeCP2 notably modulated the expression of several proteins involved in the hippocampal synaptic plasticity and learning and memory [41]. Either down- or upregulation of function of MeCP2 substantially influenced synaptic plasticity and memory function in several neurological scenarios [42]. Upon binding to the methylated CpG sites in the promoter region, MeCP2 may recruit other transcriptional factors, including HDACs [43], [44], to modify the histone acetylation and repress the transcription of target genes. It was also reported that MeCP2 may form a complex with DNMT1, the enzyme that catalyzes DNA (5′-cytosine) methylation at CpG sites in promoter regions, to maintain DNA methylation in the genome, and alteration of the MeCP2 activity significantly modified the function of DNMT1 to maintain the cytosine methylation in the promoter region [30], [31]. Phosphorylation of several serine residues (e.g., serine 421) significantly modulates the association between MeCP2 and methylated CpG in the promoter region of target genes, thus regulating the occupancy of transcriptional factors and gene expression in an activity-dependent manner [45], [46]. In the present study, postnatal maternal separation appreciably decreased the association between MeCP2 and the methylated CpG sites *via* increasing the phosphorylation of serine residue 421 of MeCP2, and subsequently reduced the occupancy of transcriptional factors (HDAC2 and DNMT1) and altered the histone acetylation and cytosine methylation in the promoter region of *Crh*. These epigenetic modifications potentially accounted for the upregulation of hippocampal CRH in the rats with postnatal maternal separation. This provided a novel epigenetic mechanism underlying the hippocampal synaptic dysfunction and memory deficiency induced by postnatal maternal deficiency.

A number of evidences indicated that CRHR1 signaling predominately mediated CRH-involved modulation of neurotransmission and response to stress [47]. In the central neurons, the primary mode of CRHR1 signaling is through G protein subunit Gs*α*, which binds to the third intracellular loop of the receptor [48]. Stimulation of CRHR1 signaling results in the activation of intracellular adenylyl cyclase, cyclic AMP and protein kinase A pathway, extracellular signal-regulated kinase signaling, and other signaling pathways [48]. Previous morphological evidence confirmed the localization of CRH in the hippocampal CA1 neurons [49]. CRH, either endogenous peptide released during stress or exogenous peptide, exhibited the effect on hippocampal glutamatergic transmission and memory in the dose- and time-dependent manner [7]. Transient (a few minutes) release of CRH usually enhanced glutamatergic synaptic strength [50], *via* increasing presynaptic glutamate release [51] and/or postsynaptic excitability [10], and primed HFS-induced LTP in hippocampal neurons [52], [53], and improved the acquisition and retention in several hippocampus-dependent tasks [54]. However, sustained (hours to weeks or even longer) exposure to CRH, *via* CRHR1, substantially reduced glutamatergic synaptic plasticity, induced the loss of spines, lowered the total dendritic length and complexity in hippocampal neurons, and impaired learning and memory in the rodents [7]. Recently a reduction of hippocampal cell adhesion molecule nectin-3, which resulting from the hyperactivity of CRH-CRHR1 signaling, was reported in the rodents with exposure to early-life stress exposure, and recovery of hippocampal nectin-3 expression rescued the detrimental effects of early-life stress on memory and spine density in adulthood [5]. The present study revealed that postnatal maternal separation induced the long-lasting upregulation of hippocampal CRH, and blockade of CRH-CRHR1 signaling significantly improved the hippocampal synaptic plasticity and performance in the memory behavioral tests in the modeled rats. These results indicated the critical role of upregulation of CRH-CRHR1 signaling in the pathogenesis of memory deficiency-induced by postnatal maternal separation.

Series of evidences demonstrated that environmental enrichment substantially improved the glutamatergic synaptic plasticity in the central neurons and cognitive performance in the rodents. Environmental enrichment significantly increased dendritic branching and length, the number of dendritic spines and the size of synapses in hippocampal and cortex neurons [13], [55], [56]. It also enhanced the neurogenesis and the integration of these newly born cells into functional circuits in the dentate gyrus [57], [58]. Enrichment induced appreciable redistribution of the glutamate receptor subunits [13] and upregulation of several proteins, such as brain-derived neurotrophic factor [59], presynaptic vesicle protein synaptophysin and postsynaptic density-95 protein [60], thus remarkably modifying the hippocampal glutamatergic transmission and synaptic plasticity. It was also shown that enrichment significantly enhanced the learning and memory in the naïve rodents [25], and ameliorated the memory defects in the rodents model of varieties of neurological disorders [13], [61]. Recent evidences also showed that enrichment significantly increased the histone acetylation [62] and altered histone methylation [63] in the *Bdnf* promoter region, and upregulated BDNF expression in the hippocampal neurons. While the maternal separation during infancy led to sustained hyperactivity of the HPA axis [2], it was reported that enriched environment significantly decreased the baseline ACTH and lowered the HPA axis responses to the mild stress, thus producing anxiolytic effect in the rodents exposed to the abused drugs [64]. In the present study, it was found that enriched environment significantly attenuated the epigenetic upregulation of CRH and recovered the hippocampal synaptic plasticity and memory function in the rats with postnatal maternal separation.

## Conclusion

It was concluded that postnatal maternal separation induced significant histone acetylation and DNA hypomethylation in the *Crh* promoter region in the hippocampal CA1, thus potentially contributing to the memory deficiency in the adult rats, which was mitigated by the sustained exposure to the enriched environment. These results provided a novel insight into the pathogenesis of postnatal maternal separation-induced behavioral impairments.
